# General Evidence in Support of the Commission

**Published:** 1852-04-01

**Authors:** 


### GENERAL EVIDENCE IN SUPPORT OF THE COMMISSION.

*Elizabeth Brown*—Lived as nurse with Captain and Mrs. Cumming for nine weeks; left on the 10th of March, 1846. Captain Cumming was at that time confined to his bed-room. Witness slept in the same room, as he required attendance at night. Mrs. Cumming slept in the back-parlour. Mrs. Cumming's conduct towards the captain was very bad indeed: one time she put her hand under his cravat, and in the scuffle the handle of the bell broke. The captain did nothing to provoke her. She used the worst of language to him. She said he whored with every one of us. Has heard Mrs. Cumming say the captain threw her money away. She would go on for a full hour at a stretch, swearing and cursing without any cause whatever. She wished God to send curses on her grandchildren. She said Mrs. Hooper went into the park with soldiers, and that was the reason she would not allow her to come to her house. She said Mr. Ince robbed her of her plate. She said Mr. Ince and I were as intimate as man and wife. The captain was very quiet when left alone. Mrs. Cumming put her money in her boots, and she slept in her boots; she was very dirty in her habits. She used to keep the cats in her bed-room, and she never allowed them to come out; they performed all the offices of nature there. Once, on a quarrel about a writing-desk, the captain raised the poker to protect himself. I was sent out; when I came back, a policeman had come in and taken the poker away.

At that time was Mrs. Cumming's conduct very violent? She was afraid of the captain just at that time. — Which had the poker? The captain had it to protect himself. The cats used to have meat at eightpence a pound: she would not let it be boiled properly; the cats used to have clean knives and forks, and plates and towels. We dare not take her a dirty one in. The meat was boiled for the cats, and the captain had the liquor. Mrs. Ince and her sister once called.

Cross-examined by Mr. James.—Was examined on the former commission. Mrs. Cumming and I were always quarrelling. Mr. Cumming was a man of gentlemanly habits. He was in the habit of calling his wife a whore when out of temper. She paid the bills. She was very careful of her money. — When she said Mrs. Hooper used to go to meet the soldiers, did not say that Mrs. Hooper, instead of going to chapel, used to go to meet Mr. Hooper, the bandsman; and did you not say that in answer to Mr. Barlow (at the former inquiry)? No answer. — Which cat was it that used the knife and fork? No cat used a knife and fork. — I thought you said so? Mrs. Cumming cut the meat. — Did you not state on a former occasion that he was frequently drunk? He could not be frequently drunk. — Was he drunk? He might be the worse for taking.

*Susan Boyd*, examined by Sir F. THESIGER.—Had been three months in the service of Captain and Mrs. Cumming. Mrs. Cumming accused her husband of having intercourse with a nurse. Witness slept in the same room as Mrs. Cumming. She was restless at night. She said Mrs. Ince was no better than a street-walker. Does not recollect that she said anything about Mrs. Hooper. Witness has seen Mrs. Cumming use violence towards the captain. Does not think she allowed him sufficient food. More dinner was provided for the cats than for the servants. Mrs. Cumming dined with Mr. Cumming.

Cross-examined by Mr. Serjeant WILKINS.—Mrs. Cumming supplied the moneys for the articles consumed in the house. She was rather near; what some people would call stingy. She often found fault with me for giving too much. She would often make purchases herself when people came to the door. They complained that she was very hard to deal with; that she knew what she was about. Mrs. Cumming paid me my wages; she did not require an acknowledgment from me; she did from Elizabeth Brown.

Did she not say that Mrs. Hooper was no better than a street-walker, for she used to pretend to go to chapel, when at the same time she used to go and meet Hooper, the trumpeter? I have often heard her say that. — Did she not say that Mrs. Ince had encouraged the marriage without her knowledge, and that she ought therefore to be ashamed of herself? Yes; I have heard her say that. — On the occasion when you and the nurse went up stairs and found her choking the captain, had she not screamed? Yes, she did. — Was that for help? She said it was for help.

The captain sometimes had turtle-soup. He had his breakfast, lunch with some brandy, dinner, tea; sometimes a little broth for supper. Will not swear that she never complained of my idle habits. When the place was kept clean she said it was not kept clean.

By the COMMISSIONER.—Mrs. Cumming was cleanly in her own habits.

*Harriet Brown*, examined by Mr. PETERSDORFF.—Resided as nurse to the captain about July 1845. Mrs. Cumming's conduct towards her husband was unkind. She was often very violent, very abusive about the cats. — Have you heard her say anything about her grandchildren? Not anything particular, except about one little one that had died; she said that Mr. Ince had glazed it over after its death to make it look like life. I could not enter into the exact words, because it did not concern me.

Cross-examined by Mr. JAMES.—When did she speak about Mr. Ince's child?—did she say that it looked beautiful, almost like a varnished wax-doll? I think I might venture to say she said that. — Did she say it looked beautiful? No, she said the child looked as if it had been glazed over; like those children or wax images you see in tailors' shops. I was there from the 10th of July to the last day of August in 1845. — The captain used to have some lunch I suppose? He used to have some beef-water. — What is beef-water? A pound of beef was ordered; it was sometimes boiled not more than ten minutes.

Mrs. CUMMING.—That is a gross falsity.

*Sarah Allsopp*, examined by Sir F. THESIGER.—Formerly lived for fourteen years in service of Mr. and Mrs. Ince. Knew Mrs. Cumming. Went with Mrs. Ince to visit Mrs. Cumming in 1837. At that time Mrs. Cumming was sometimes affectionate, and sometimes I thought her very excitable and very indifferent to her daughter. Went with Mrs. Ince and the baby to visit Mrs. Cumming at Maida Vale in 1840; her conduct was rather more strange. Mrs. Cumming and Mrs. Ince called once together upon Mrs. Hooper.

Cross-examined by Mr. JAMES.—Have you not heard that Mrs. Cumming was kind towards the daughter, and that she never liked Mr. Ince at all?—I am sure she never did like Mr. Ince. Was never present when Mrs. Hooper was present with Mrs. Cumming.

*George Vernon Driver*, examined by Sir F. THESIGER.—I am a surgeon. From the year 1840 to 1847 I was in the establishment of Mr. Ince; I was on intimate terms with Mr. and Mrs. Cumming. I visited Mrs. Cumming on two or three occasions with Mrs. Ince. Mrs. Ince was very dutiful and very kind. I first remarked Mrs. Cumming's conduct to her daughter at an evening party at Maida Vale; a circumstance occurred which caused a little earlier separation than otherwise would have been. I was seated very near Mrs. Cumming and Mrs. Hutchinson, and I overheard something said disrespectful of the captain. It brought the remark afterwards, "The old fool is as deaf as a post." Mrs. Cumming checked the remark, and

I heard her then say, "The old fool is not so deaf but he can hear what has passed." That excited attention, and the party separated in consequence. Before that circumstance I had observed nothing extraordinary in the conduct of Mrs. Cumming, either to Mr. Cumming or her daughter, Mrs. Ince. — After that time, when you visited with Mrs. Ince, did you observe on any occasion the conduct of her mother to her different from what it had been? No, I observed nothing different from what you would expect. Latterly, Mrs. Cumming's conduct towards her family became more and more estranged. She did not pay proper attention to Mr. Cumming. On one occasion, about six months before his death, the captain came to Mr. Ince's in great bodily fear, he said; he was filthy in the extreme; he had large ulcers about him; Mr. Ince changed his linen; he was then removed to Belgrave-place. She once took me to see her fowls, and in my presence she made water, without offering any remark. (She has paralysis of the bladder.) The house was in a very noxious state.

Cross-examined by Mr. Serjeant Wilkins.—I am on intimate terms with Mr. Ince. I live within a few doors. At the time of the last commission I was living with Mr. Ince. I did not give evidence then.

*Mr. Thomas Ince.*—A cousin of Mr. John Ince, the son-in-law of Mrs. Cumming. Has known Mrs. Cumming three or four years. Knew her before Mrs. Ince's marriage. When I first knew her she was very lady-like in her manners, very neat in her person. I should think she had received a good education. She behaved to her daughters very well indeed; in the usual way. That was seventeen or eighteen years ago, before the marriage. — How did she behave at that time to Captain Cumming? I so rarely saw her that I cannot exactly say. — You had not an opportunity of observing? No, sir.

Shortly before the marriage of Mrs. Ince she said that her daughter (Catherine) had been the best of daughters, and would be a very great loss to her indeed. I knew Mrs. Hooper. After her marriage there was some estrangement between Mrs. Cumming and her. I do not know of the intimacy being at any time resumed after the marriage. I did not at any time notice a difference on the part of Mrs. Cumming towards her family. I continued to visit her for about two years after Mrs. Ince's marriage. I am now on terms of intimacy with the Inces. I have not known of any acts of unkindness or harshness on the part of Mrs. Ince towards Mrs. Cumming.

Cross-examined.—I have not seen Mrs. Cumming for twelve or fourteen years. I cannot exactly say that I was very intimate, but I visited her. I should say I did visit her more than four times in the four years. — How often do you believe you visited her during that time? I really cannot say. — It is merely a question of memory. There are a good many doctors here? My memory is getting rather bad. — Now you have been asked whether you know anything which should have created an aversion on the part of Mrs. Cumming towards Mrs. Ince? Yes. — Were you aware of the taking out of the Commission of Lunacy in 1846? Yes. I am not aware of any of the circumstances which have occurred since 1846.

*Mr. Dangerfield.*—I am a solicitor. Mrs. Cumming applied to me in the beginning of 1844. She had previously employed Mr. Hawkins as her agent. Various proposals were made to her for the letting of the Stow Hill House. It was in a very wretched condition, and I recommended that something should be done with it. She had always some objections to make. I never could get any decided answer from her. I think latterly she seemed inclined to entertain some proposals. There were various complaints made from tenants and other parties, and I found that it was necessary that there should be a general survey of the estate. I sent my brother down in the spring of 1844. He made a very full report of the state of the property; a copy was given to Mrs. Cumming. I do not particularly recollect that she attended to any of the recommendations which were contained in that report. She always expressed herself with great gratitude for all that I had done for her. I recollect she came to me in the beginning of October 1844, when she was about to take a furnished house at Bayswater. I found she was in rather distressed circumstances. I pointed out to her that if she had two houses she would increase her difficulties, and that if it was possible she should not take this house. I think that was the last time I saw her. I had rendered to her my accounts from time to time as I received them. I paid her over the money. I have not my books here of my attendances and bills of costs. I have still a claim for costs. I never robbed Mrs. Cumming out of 200*l*., or any other sum. I could not rob her of any plate, for I never saw it. During the time I was her solicitor she always spoke very slightingly of Mr. and Mrs. Ince and Mr. and

Mrs. Hooper, and did not wish that they should be acquainted with her address. She always spoke of Hooper as being a person rather inferior to her position.

I compromised an action brought against her for misusing the furniture in a furnished house. There was one bed in that house she said she would not sleep in, because they had weighed the feathers.

I was the solicitor employed upon the Commission in 1846. The order for the confinement of Mrs. Cumming was signed in May. I understood it was signed by Captain Cumming. She was detained in the York House Asylum; then I was informed of it; and it became important to ascertain whether the Commissioners would, under the powers of the Lunacy Act, which enables them under certain circumstances to do so, appoint a receiver of the estates without a commission. The Lunacy Commissioners made inquiry into that; they satisfied themselves I believe that Mrs. Cumming was insane, but the Commissioners thought it was only intended to apply to small estates, and therefore that it was not desirable in this case to act except by a Commission. The matter was delayed in consequence; it was going on at the time of Mr. Cumming's death. After that the Commission was issued at the instance of the two sons-in-law and their wives. After the Commission had sat some days there was an arrangement come to by counsel on both sides.

Mr. James here said—It had the sanction of several of the jury; if it had not I should not have acceded to it.

The Commissioner.—What did take place was this: an agreement was entered into which was mentioned to me, but I declined to go into it at all, and I think the counsel on both sides felt professionally that there was legally a little difficulty about it. It was put to me, whether I could not, in the phrase of the Common Law Courts, allow a juror to be withdrawn, or, what could be done. I said I felt there was great difficulty as to any course that was to be pursued for any such purpose, but I expressly protected myself from giving an opinion. It was clear that, the Crown having issued a Commission, it was to be proceeded with or dropped, or some course taken, and there was no possibility of either party withdrawing a juror. I did what I thought was the legal and proper course—the case was adjourned. Some suggestion was made by counsel, that it was for me to say what course I thought fit to pursue, and on the following morning I asked counsel on the one side and on the other side to proceed, and they declined, and then I said, under those circumstances I felt it was impossible for me or the jury to conduct such an inquiry; and I asked whether either party asked for a verdict; they said, no. I said under those circumstances I could not go on with the inquiry. I had then no alternative but to dismiss the jury, and the Commission was returned with an endorsement, stating the terms of the compromise.

Cross-examined.—When did you render to Mrs. Cumming an account in writing of the rents you received for her?—The accounts I rendered were verbal. I do not know that I even rendered a written account, except upon one occasion. I have heard that Captain Cumming took the benefit of the Insolvent Act, about the year 1837.

I ask you if you do not know that this house which you have been telling the jury this old lady allowed to go into decay was scheduled in 1844 for the improvement of the town? I have heard that a water-works company was in progress.—You do not know that the house has been taken? I think I have heard that some such act passed, and that the house has been sold. Mr. Ince and Mr. Hooper first consulted me about the Commission in the spring of 1846.—In the first instance, was not this lady taken to York House Lunatic Asylum on a certificate signed by Mr. Johnson only, who was a friend of Mr. Ince's?—I have heard so. I was at her house in Belgrave-place before the inquiry, and while she was in custody at York House, with Mr. Ince; whether Mr. Hooper was there I do not know; he might have been. I have no recollection of helping them to find the property in the house. I recollect looking into some cupboards and places. If any property was taken away it must have been something very slight. I have no recollection of any trinkets. I had nothing to do with the return of any property under the arrangement, except as to the deeds in my possession. I delivered up also all the various papers of Mrs. Cumming, independent of the terms of that arrangement.—Had those been taken from her while she was in the asylum? I have no doubt. I tell you I had them from Mr. Hooper, and I blamed him for not bringing them before. I acted as the attorney for Mr. Ince and Mr. Hooper for a very short time after the arrangement, until Mrs. Cumming repudiated it.

Re-examined.—Visited the house in Belgrave-place for the purpose of preparing brief for counsel for the commission in 1846. There was a view of the jury in consequence. It was a very extraordinary exhibition; in an upper room the filth of the pigeons was of enormous depth; and the furniture and the things lying about, and the way they were packed up, was extraordinary altogether. [It must be observed, that this refers to a period four months after Mrs. Cumming had been carried away to the asylum.]

*Winifred Todd*, examined.—Lived as cook to Mrs. Cumming, at Gothic-villa. Went on the 13th of June, 1850. My niece and myself were the only servants at the time. Three or four days after I had been there Mrs. Cumming asked me to lend her a penny. From time to time I advanced her money to pay for things that were required in the house, to the amount of about thirty shillings; and then she gave me two pounds, and I gave her back ten shillings. She always seemed deeply affected that she could not have the control of her money. It was on a Thursday Mr. Haynes said he would take her to Orchard-street, to Mr. Robinson's, to receive some money. Mrs. Cumming was dressed all but her bonnet. When Mr. Haynes came up she said, "This is a pretty start;" and he said, "What is the matter?" She said, "I have not got a bonnet to go out in;" and he replied, "Where is the bonnet you travelled in from Wales?" she said, "That bonnet I cannot find." I searched the boxes and could not find it. Mr. Haynes said, "Never mind, Mrs. Cumming, I will see into it immediately." The following day I found the bonnet was then in the house. She said that she had been robbed of her knives and forks. Mrs. Cumming was in the habit of giving orders first for one thing and then countermanding them. She has gone frequently the whole day and night without anything except a little brandy and water and a little port wine. I was three months the first time, and two months the second time, with Mrs. Cumming. I was six months away from her. She came in one time at one o'clock at night; she had ordered dinner at six o'clock; she had her dinner at one o'clock in the morning. I have set up with her at night. She was in the habit of swearing when she used to be vexed about her money. I heard her say that Mr. Haynes said it would be better for her not to see her children, as they were the means of her being locked-up before, and of course they would be the means of her being locked-up again. I heard her say that every servant robbed her.

Mr. Serjeant WILKINS interposed here.—There is not one single answer that this witness has given you that you did not put into her mouth.

Mr. PETERSDORFF.—Then now I will alter it.

She would sometimes dirty the bed. She seemed sorry at times that such things had occurred. At other times she did it on purpose of aggravation, because I wanted to go down stairs.—Did she say anything on these occasions about murder? I never heard her say anything the whole time I was there about murder.—Was anything said about striking or knocking you? She never said anything of the sort to me. She had things cooked in her room, except it was a joint, and then she had it cooked in the kitchen. She would frequently order different things and then refuse to eat them.—Give us some instance when she ordered inconsistent things? Sometimes she would have a slice of salmon, when she got it her stomach would not take it, and then she would have a chop; and then she would not have that; and then a stewed eel, and then her stomach would turn, and she would not be able to take anything. She had four cats. They generally lay in the room where she was. It was at times very offensive. There was a convenience put underneath for the animals to go to. She seemed very deeply affected that Mr. Robert Haynes had denied her money, and she said that she had got none, and that she would come to the workhouse. She sent me to Mr. Passy, a silversmith, to learn if he could recommend her a solicitor. He recommended Mr. Thorn. Mr. Thorn came on the following evening. She applied to Mr. Thorn for money. The cats always had their plates, and their meat and soup every day. There was a cloth laid generally for them. They always had their milk in a china cup.—And saucer? No, only a cup. Dr. Caldwell attended her. The medicine was always put away when he was coming. Dr. Caldwell did not know anything about it. I heard her say that a person of the name of Clark had got some person to poison her; that she had given the milk to the cat, and that it was analysed by Dr. Hale. It was sent over to the chemist; that he said it was poison. —Do you remember her saying anything about her own food? No, I never heard her say anything, except that she liked to have it cooked in her own room, and then she knew what she was eating. She would not touch some fruit from Mrs. Hutchinson, for she believed she was the biggest enemy she had. — Do you know whether she said anything had been done to the fruit? I do not recollect her saying anything about it. The house was dirty at times, because it was up stairs. My niece did not keep the room up stairs so nice and clean as it ought to have been kept. I never saw any one visit her but Mr. Haynes, Miss Cook, Mr. Fischel, the two Misses Hutchinson, and Dr. Caldwell. Miss Hunt called once. Mr. Haynes came about half-a-dozen times. He came when she sent for him.

Cross-examined.—Do you not know that her affliction was such that she could not restrain her stools? Sometimes she could if she chose. — On many occasions did she not express sorrow after it? Sometimes she has. — I think you gave the name of the doctor of whom she spoke about the poison as Dr. Hale; recollect yourself, was it not Dr. Barnes? No, Dr. Hale. — It is quite impossible you could be mistaken? No, I might be mistaken. — Now, is it not a fact, that while you were there some person did attempt to rob the premises? One night I was up with Mrs. Cumming, and all at once I heard a noise down stairs; when I got to the top of the kitchen stairs the wind nearly blew the candle out, and I found the door open. I saw a man go up the steps, and in the morning I saw the bar was taken out of the window, and the window stood open. I named it to Mr. Haynes. I did not name it to Mrs. Cumming till the Sunday. When I went to the house the kitchen had been stripped of almost everything. A box of sand was kept under the bed for the cats. — Where was it that the bonnet was found afterwards? In a box with another one, where she had put it and forgotten it. — You say she often changed her intentions about her dinner; she was in that sickly squeamish state that she could not relish anything hardly? No, she could not.

Re-examined.—Did Mrs. Cumming ever say anything to you about the want of the different things in the house? how it came that she was without these things? She said she had had a great many servants, and she supposed one had robbed her, and another had robbed her; she could not get down stairs to see after them.

*Mary Rainey* examined.—I was in the service of Mrs. Cumming in the latter end of November. — In what year? I cannot say what year it was. — Was it last November? It was. — Was it the last November, or the November before the last? How long is it since you left the house? Last November twelve months. — Then it was in November, 1850? I do not know what the number of the year was. She was then living in the Queen's Road. I lived with her upwards of two months. I left her on the 2nd or 3rd of February. Only Mrs. Cumming and I were living in the house. On the morning of the 1st of February I recollect the police coming. About twelve o'clock at night Mrs. Cumming got excited, and she is a very helpless person. Mrs. Cumming could not get from one side of the room to the other without I assisted her, and on this night in question Mrs. Cumming walked round the room as nimbly as you or I could, and she came to the window, and I asked her what she wanted. Then she got outside on the landing, and I asked her what she wanted, and she said it was no matter to me what she wanted; I was to mind my own business. She threw up the staircase window, and I turned her away from the window. I asked her what she wanted at the window? and she said I had nothing to do with that. Shortly afterwards she asked for some supper, which I brought to her. — Go on; what then? we are not aware of all these things. — I know what happened; you have got my statement before you; I dare say you know the questions; you have them before you, but I have not. — I want to know what happened. — I want to know too. — What then did she do? You must ask me the questions and I will answer them. — Do not make a joke of this. Recollect if you please you are upon your oath, and you are to tell us the truth. What did she do after her supper? (The witness here entered upon a matter totally irrelevant to the question.) Now, if you will apply your mind to what occurred on the 1st of February; do you remember the police coming? Yes; Mrs. Cumming had been excited, she locked herself into her room, and was calling me. I had removed the knives out of the room the night before. She threatened to cut her throat; she said she would put an end to all this; then she asked me to go down stairs and fetch her some coals. I went, and during my absence she locked herself into her room. I had taken the key out of the bed-room door, but she bolted the door inside, so that I could not get in. I tried to get in; she said I should not get into her room. The coachman, Charles Crane, (see his evidence) and I tried to get into the room. Mrs. Cumming was not aware that he was in the house. I found that Mrs. Cumming was speaking to the police outside in front of the house. Before she was speaking to the police I

heard a cry of "murder." I went down stairs, and asked who was there, and he said "The policeman." I opened the door to the policeman and to another, and left one outside, and she threatened to me, as she had threatened before, to throw herself out of the window. She said there were a great many ways in which she could destroy herself. The policemen went up stairs to her door, and afterwards they got in. They could not get in by the door. They told her who they were. There was a conservatory leading into one of Mrs. Cumming's rooms; I told the policeman if he got into the conservatory, by a chair and table, he could get into the room. He got in in that way. The coachman broke the window, and the serjeant of the police entered Mrs. Cumming's room; he then opened the door and allowed the policeman and I to go into the room. The policeman stumbled; she screamed out; I could not say whether it was murder, but she swore at him for getting through the window. He said to her "I am come to protect you." She said, "You are not come to protect me; you are a person in disguise come to take me to the madhouse." After a very short time the policeman left. Mrs. Cumming was quiet after the policeman went that night. I afterwards sent the coachman to Mr. Hutchinson's; I asked him at the same time to call for Mr. Thorn, and he told me he would do so, and he did not do so. — I asked you whether you desired him to go to Mr. Thorn? It is written in my statement, and I must state it to you, must I not? — We have a great deal to do. So have I. Mr. Haynes came on the same evening. When he came, he was shown into Mrs. Cumming's bed-room, and he asked the old dame "what was up now?" — Were you present at the time? No, I was not. I went for Mr. Thorn by Mr. Haynes coming there. Mr. Thorn was her solicitor, and I thought it was my place to go there and tell him; that was on Monday or Tuesday; when I came back I was shut out. On the Sunday night, Mr. Haynes had sent a man named Clarke and his wife. That was the way I left Mrs. Cumming's service. She had five cats; they were kept continually in Mrs. Cumming's room; it was in a dirty filthy state. I slept in Mrs. Cumming's room; I have felt it ever since, and I dare say I shall till the day of my death. She would not allow the room to be cleaned. In the drawing-room underneath where Mrs. Cumming slept, there was the wet came down through the ceiling in consequence of the cats being kept there. I dare say the stains are on the boards now, or there have been new boards. I do not think anything else could take the stains out, they were such filthy stains. The dinners were dressed in her room, except three joints. The cats had a medical attendant; his name was "Dr. Williams." Dr. Caldwell used to find the room offensive; he wished to have the window and the door open, and she told him he wanted to kill her like all the rest of them. Whenever any stranger came, whatever passion she was in, she would sit down and smile as if nothing had happened. She would sit up till four or five in the morning talking about her property and her family, and Mr. Robert Haynes robbing her, and about Mrs. Hutchinson. Dr. Caldwell sent her two bottles of medicine every week. — Was Dr. Caldwell aware that she did not take the medicine? He could not be off it, because the bottles of medicine were placed on the mantel-shelf before him. I was not aware of the address of Mr. Ince till after I had left Mrs. Cumming. — Do you recollect anything about poison in milk? Mrs. Cumming said she had some milk, that it was analysed by Dr. Hale and Mr. Ingram, and that there was found a small portion of arsenic in it. Mr. Haynes had sent her about two dozen and-a-half of wine; she said it was like bog-water; that it was nothing but poison, and that she sent it back again. Mrs. Hutchinson sent her some grapes, and she would not accept of them; she was frightened of being poisoned because she was connected with Robert Haynes. Mrs. Hutchinson, Dr. Caldwell, and Miss Hunt were the only persons who visited her while I was there.

Cross-examined.—When I first went to Mrs. Cumming, Mrs. Todd and her niece were there. I introduced into the house some persons of the name of Hickey. I am an Irishwoman. I do not know that the Hickeys are Irish. The mother introduced her daughter. I did describe myself in my affidavit as "extremely mild and not of a quarrelsome disposition." — How did you remove Mrs. Cumming from the window? By turning her round by the arm. — Of course in an extremely mild manner? Yes; that I will swear; I could not wrong my conscience to do so. — Now I ask you, if the coachman did not go up stairs, and when he went into the room whether the table in the bed-room was not thrown down, and whether Mrs. Cumming did not charge you with ill-treating her? No; certainly not. — That you swear? Yes. — Do you remember Mr. Haynes coming there? Yes. — Did Mrs. Cumming charge you with ill-treating her? No; she did not. — Was not this shawl produced, and did not Mrs.

Cumming say that you had tied this round her, and tied her arms in Mr. Haynes' presence? No.—Did you not fold your arms, and stand in the room and say, that you had done it, and would do it again? I said that if there was a doctor there from the asylum she would soon be bound over.—What had you threatened to tie her down with? I do not know. She asked me what she could be tied down with? and I said she could be very soon secured; "very likely I could secure her."—Then you did talk to her about tying her down with the shawl: I said so; I told you that over and over again.—Did you tell her so? No I did not.—Just this moment I understood you to say that you did tell her you could easily secure her. I say what I did say.—Did you tell her you could secure her? No.—Upon your solemn oath did you not say so just now? *I told her I could very soon secure her.*—What with? I do not know what with.—Did you not tell her with a shawl? *I told her I could use the shawl.*—Was it when the police were in the house that you told her you could secure her? After the police left.—I thought you said she was perfectly quiet after the police left? Yes.—After the police had left, and she was quiet, you told her you could easily secure her? She wanted to get out of the house, and I said, "If you do not keep quiet, you will be settled very soon."—You made a statement to make an affidavit in Chancery of *all the facts* you know? Yes.

The COMMISSIONER.—Did you tell Mr. Turner all the facts you knew? Yes.—(The witness being asked to read her affidavit, said she could neither read nor write. It was read to her; when it appeared that it contained no reference to arsenic being found in the milk.)

By Mr. JAMES.—When did you suggest for the first time about the wine sent from Mr. Haynes being poisoned; there is not a word of it in the affidavit? I think it is since; I am not certain.—Who is this Dr. Williams, the doctor who attended the cats? I do not know where he is; if you look in the *Court Guide* you will find it.—You never saw him at all? No; he only sent his bill in.—Is Hickey here to-day? I do not know.—When did you last see her to-day? I saw her outside.—Did you not tell the coachman that you knew Mrs. Cumming was mad directly you saw her? Yes; and so she was.—You knew it directly you saw her? Anybody would know it.—And you made up your mind directly you saw her that she was mad? No. I was discharged on the 3rd of February. I think I went to Mr. Ince's on the 4th or 5th.—Have you seen much of Mr. Ince lately? Mr. Ince?—Yes; that is what I said. Yes.—Have you seen him frequently? No; I met Mr. Hooper at Mr. Ince's. Mr. Turner was there; I made my statement there.

*James Richards* examined.—I am a policeman. In February, 1851, Howley-place formed part of my district. On the 1st Feb., about half-past twelve, I heard screams from Herbert Villa. 56 D came up at the time. Mrs. Cumming threw open a window on the first-floor front. She screamed for some minutes before the window was thrown open. We showed ourselves. Mrs. Cumming said, "Who is there?" I said, "The police." I asked her what was the matter? she said her servant was going to kill her. I said, "Can I come in?" She said, "Yes, my servant will open the door." I rang the bell and the servant opened the door. I asked what was the matter? She said her mistress was in a state of insanity, and that she had fastened herself into her room and she could not get access to her. She stated that there was a large fire in her room, and she was afraid Mrs. Cumming would burn herself. She then showed me and the other man the door leading to Mrs. Cumming's room. I knocked at the door; she said, "Who is there?" I said, "Police;" she said, "What do you want?" I said, "I am come up to interfere respecting your servant who is going to kill you." I asked of Mrs. Cumming whether she would give the servant in charge, she said, "Oh, yes, take her away." I said, "I must see you personally before I take the charge;" I said this to get Mrs. Cumming to open the door; she said, "I cannot, I am undressed." Finding that she would not open the door, I persuaded the coachman and the servant-maid to break open the door. He attempted to break open the door with an iron bar. Finding that the door would not open readily, we got on the zinc flat at the top of the staircase. The coachman broke the pane of glass in the window at the end of Mrs. Cumming's room. I then unhasped the window and got in. Mrs. Cumming seemed frightened and screamed very loud. She then went to the front window, I believe with the intention of jumping out of the window. I endeavoured to calm her. Policeman D. Parsons was standing outside: she said to him, "Who is that?" The constable said, "I am a policeman." She said, "You are an infernal liar, you are not a policeman, but a keeper from the madhouse in disguise, come to take me away." I

then opened the bedroom door and let the constable in and the servant-maid. The coachman said, "Do not let Mrs. Cumming know that I am here;" but soon after that he showed himself to Mrs. Cumming. She asked him how he came there. I saw a cloth laid on the floor, and I smelt a bad smell. I left Mrs. Cumming in charge of her servants; she seemed to be a little pacified.

Cross-examined.—I never was in Mrs. Cumming's house before. Did not know Mary Rainey. — When the window was open, could she have thrown herself out if she had liked? She could. — She was in the room by herself? She was. — You got into the lady's bed-room? Yes. — And first she peaceably asked you what you wanted? She did. — Did you happen to stumble and fall head-foremost? Unfortunately I did.

*Arthur Parsons* examined.—A policeman. I was on duty on the 1st February at Herbert-villa. I remember Mrs. Cumming throwing open the window, but not the word "murder." I heard some sort of scream. (This witness generally gave the same evidence as the preceding).

*Eleanor Hickey* examined.—The wife of a plasterer. I was introduced to Mrs. Cumming either the last week in November or the first week in December, 1850, by Mary Rainey, one of the witnesses who has been called. I took my daughter Mary Ann, who was then twelve years of age, with me. She asked me if I would like her to come out to service. I arranged that my daughter should go. She asked Mary Ann if she was fond of cats. She said "They are beautiful creatures, I will tell you their names;" the first was called "Vic," the second was "Viz," the third "Mrs. Thomas," the next "Kitty," another "Tommy;" those were the quantity that were in the room at the time I knew Mrs. Cumming. My daughter was there about seven weeks. I arranged also that another daughter, Ellen, should go into Mrs. Cumming's service about the week before Christmas (1850). She wished to engage Ellen as housemaid. She was going to remove to Howley-place. She said the house must be kept more cleanly than the one she was in then, by which means she must have an extra servant. I heard complaints about wages not being paid. I have heard a statement about Mrs. Cumming having taken a dislike to her daughters. I went to see Mrs. Cumming when my eldest daughter had been there about a week. — Will you state what Mrs. Cumming said to you, or you to her, when you saw her? When I first came, I went into the kitchen and took a small portion of spirits, which I always did before I entered her room, because the smell was so truly obnoxious that I could not stand it. She was sitting by a very little bit of fire in her dressing-gown, which she generally wore extremely dirty. She said my daughter and the "mistress of the kitchen," meaning the cook, had kept her without a bit of food all day; that they would not answer the bell, nor put a bit of fire on. I offered to fetch her something; she says, "My appetite is quite gone; but were it not, I cannot afford it." My daughter came up. Mrs. Cumming said, pointing to the cat's table on the floor, "Look, Mrs. Hickey, those four animals have got dirty plates." I told my daughter to take the dirty plates away and bring clean ones; and Mrs. Cumming was very much pleased. I visited Mrs. Cumming again three days after; she said to me, "I have signed that cursed will in Mrs. Hutchinson's parlour, so they tell me, but it is not my will, and I shall send for Mr. Thorn, a most respectable gentleman." The ceiling of the drawing-room was nearly falling through with the filth from the cats, and on the carpet and ground. I drew her attention to it, I said, "If Hickey has this to do he will have to cut all this ceiling out." I went to her after her removal to Maida-hill West (to reside with her). (Witness described a dirty act of Mrs. Cumming, which she represented that Mrs. C. had done expressly to annoy the servants). I dressed her on one occasion to receive Sir Matthew Wyatt, who was to come to sign different agreements about the house. After she was dressed she said, "Don't I look nice? because you know, Mrs. Hickey, Sir Matthew is coming as a courter." I says, "Sir Matthew is married, Mrs. Cumming;" she says, "Oh dear me, no, it is a mistake." I stayed there five days. The first night I did not sleep at all; I was apprehensive of something happening to Mrs. Cumming, from what I had observed myself. Mrs. Cumming said she was confident there would be something happen to her. She would sit up at night and say there were persons coming about to destroy her, and take her to a madhouse; her children were pursuing her, and trying to get her confined, that they might take her property from her. I left my daughter Mary Ann there. I went again to fetch my daughter away. I am convinced there was no complaint against Mary Rainey. One time I went and could not get admission—a Sunday evening. I called for a chisel and hammer of my husband's. Mary Rainey spoke to me at the door, she said

"Things have taken a very serious turn." I went again on the Monday morning. A man named Clarke came to the door; he brought me a chisel. The next time I went I asked for the hammer. I think it was two or three evenings after I went again, and saw three vans at the door. I waited till Sir Matthew Wyatt came to the door. He made some inquiries of me. I got a cab and went to Mr. Ince's. I saw Mrs. Ince. I then went to Sloane-street, to a Mr. Jones. Mr. Ince was there. I stated to Mr. Ince what had happened. Mr. Ince and Mr. Hooper went together to Sir Matthew Wyatt. I knew where Mrs. Ince lived before this. I had inquired who were her relations.

Cross-examined.—How many of the Hickey family were there in the house? My two daughters and myself. Mary Rainey was there all along; she introduced the Hickey family. I was in the house five days. Two or three days after I was there I looked into the *Directory* for the Inces' address. — I suppose your curiosity was a little excited? Not at all, it was quite a different feeling. — What was it? It was a feeling that any mother would have towards a family, and upon public grounds. — Did you go down to Mrs. Hutchinson's when Mrs. Cumming was arrested by the police on a charge of perjury? Yes. — Were you there? At what time? — I will tell you; you went down to identify Mrs. Cumming when the police arrested her? Yes, I went to Stamford-street. — I believe you went to identify her? I did not go particularly for that, I went to Mr. Hutchinson's house for my husband's hammer—for that very day he was going to work. — What I was asking you was, whether you went down to identify Mrs. Cumming in order that she might be arrested? I did not. — You swear that? *Not particularly for that*—what I went particularly for was my husband's hammer. — Attend to me—upon your solemn oath, did you not go down to identify Mrs. Cumming to be arrested by the police? Not when I left my own home to go there; but when I got to the office, they asked me there if I knew Mrs. Cumming personally, I said "Yes, I do." — What office? At Stone End police-office. — What took you there—was your hammer there? You will not allow me to explain where my hammer was. Yes, gentlemen, you may laugh, certainly. When I went for the hammer, Mrs. Hutchinson treated me very unlike a lady, and said there was nothing there belonging to me, and that Mrs. Cumming she knew nothing of. I said, "If you do not give me my husband's property, or tell me where to find Mrs. Cumming, I will compel you;" and as I went out I thought I would go down to the Stone End court—*there was a case coming on there, and I met a gentleman named Jones.* He said, "Where are you going, Mrs. Hickey?" We went to the Stone End court together. *I did not go in.* When he came out, an officer came and said, "Should you know Mrs. Cumming, provided you saw her?" I said "Yes." He (Jones) said, "You had better go down with this officer, because they want somebody to identify her—they are going to take Mrs. Cumming upon a warrant." When we went down, there was a clerk from some office. The officer knocked at the door, and the servant opened, and we all went in. Mrs. Hutchinson came to the door, and said, "She is not here." I said, "You know she is here—officers, I am confident she is here." The officer then said, "To tell you the truth, I hold a warrant, and you must allow me to search the house." She said, "Wait a minute:" she went to lock the door; there is a middle door in the passage; I saw that her intention was to lock the door, and said, "*Officers do your duty, and proceed up stairs.*" — You went to identify her? At that time. — Were you in the drawing-room with the police when she was arrested? Sir? — You heard me? *I was;* but the door was not open. — Were you in the drawing-room when she was arrested? No; the door was not open during the time I was there. — Were you in the drawing-room? *Yes*, and the old lady was in her bed-room. — Were you in the drawing-room with the police? *Yes.* — Did Mr. Haynes order you off the premises? Mr. Haynes asked me what I wanted? I said, "Sir, I have an interest in the case." — What Mr. Jones was it you saw about this arrest? Mr. Ebenezer Jones. — Had you seen him before? Yes. — Where had you seen him? I will not be certain. — I think I can inform you. I cannot exactly recollect. — Where have you seen him? I think the first time was at Mr. Ince's. — How shortly had you seen him before you went to identify this lady? Two or three days. — Where? Am I compelled to answer that question? — If you wish to be believed. At an attorney's office in the city, Mr. Turnley's. — Was he the man that went with the police to arrest the old lady? *No.* — Did you not say you met him? Certainly. — Is that the Mr. Jones you met about the police to identify the old lady, that you met at Mr. Ince's? *Yes.* — Did you see Mr. Jones after that? I saw him the following day—he called on me at my place; he called to speak to me about Mrs. Cumming, and about the affair. (Witness at first refused to say what he came about, then said): Jones said there was a case coming forward against Mrs. Cumming and Robert Haynes that would very much surprise me. — Did you see Mr. Jones with Mr. Hooper? Never but once, since I came here—they were walking about together. — I thought you said that when you took Mary Ann you were introduced to the five cats; when you were introduced to those cats, the place was filthy dirty, and smelt very bad? Yes. — And you left your daughter there notwithstanding? Yes. — Three weeks after you sent another daughter? Yes. — Were you at Mr. Rumsey's more than once? I do not think I were, but I cannot charge my memory with that. — What took you to Mr. Rumsey's? I think I went there with Mr. Jones (Ebenezer). — Have you the slightest doubt about it? I am a person that has a little family to see to, and I do not bother my brains about all these sort of things. — Did Mr. Ebenezer Jones take you to Mr. Rumsey's? To the best of my knowledge, I believe he did. — Were you, besides being at Mrs. Hutchinson's to identify the old lady when she was arrested, were you before the magistrate, Mr. Gilbert A'Beckett, at Stone End? I was in court, but was never questioned. — You were there at the time you were about the hammer? Yes; but I never got the hammer.

*Mary Ann Hickey* examined.—The daughter of the last witness. Went into Mrs. Cumming's service about three weeks before Christmas. I was principally in the bed-room. Mrs. Cumming took all her meals there. I read the newspaper and attended to the cats. The cats had napkins and plates. I used to cut up their victuals. She has threatened to cut my throat about the cats. I was cutting up the cat's meat, and she said I was not doing it properly; she said if I did not mind, she would cut my throat. She seemed angry; she had a small dinner-knife in her hand. That was said in a manner that frightened me—I thought she meant it. — Do you remember at any time her being angry about some knives and forks? Yes; she was having some dinner, and she asked me to bring her a knife and fork, and I took her a fork with two prongs; she said she did not want that, and I took her the basket. She took up a small-handled knife, and she said, "It looks very tempting, don't it?" She says, "If you don't mind, I will cut your throat with it." I ran out of the room. I did not think it was joke, she ground her teeth together. The windows were never opened. She would not have the door opened if she knew it. All the filth of the cats was left under the bed. There was a box underneath the bed, nearly half full. I could not see whether there was any *sand, because it was underneath.* Mrs. Cumming herself was dirty in her person. — Do you remember ever hearing Mrs. Cumming say anything about doing anything to herself? I have not heard her say so. She swore very much. There was no cause for her being angry.

Cross-examined.—I am thirteen years old. — Your mother having been five days in the house with you, and seeing all that was going on, she left you there as servant? Yes; I had not been there long before Mrs. Cumming wanted to see my sister Ellen. My mother went away after stopping there five days, and left us both there. — When was it Mrs. Cumming talked about cutting your throat? After I had been there about two weeks. — Was it before your mother came to stay the five days? Yes. — When your mother came to stay, did you tell her? Yes. — What did she say? She took me away, and then Mrs. Cumming wanted me again. — Before she took you away had she sent the other daughter there, your sister? Yes, that was after I had told her about cutting my throat. I was not very frightened then. I remember Mary Rainey being there; she and Mrs. Cumming were always quarrelling. — Am I wrong in imagining from my learned friend's examination that each cat had a table-napkin? There was not while I was there; the cloth was spread upon the carpet, and then the meat was put upon the cloth, and a cup of milk by the side of each plate. Mary Rainey did not like the cats, because she had to clean the room.

Re-examined.—The cats did not have a clean cloth every day; once a week.

By the Commissioner.—In Herbert Villa there was no box under the bed; the mess was cleaned up as they did it. I went back willingly to live with Mrs. Cumming. I was not very frightened when I went back. — We have been told that once or twice you laughed at her? Yes; she used to shake her hands about, and I could not help laughing. — Did you ask your mother to take you away? No; but Mrs. Cumming said she would not have me in the house.

*Ellen Thomson* examined.—I am the daughter of Mrs. Hickey. I am just turned sixteen. I was in Mrs. Cumming's service about three weeks, at the Queen's-road. The second day I was there she said I was a d—d little defrauding ——. I had done nothing to offend her. She set me to clean the cat's soil which was underneath the bed. While I was there I went up several times and cleaned it away. Once she ordered me to bring up some plates. She took one of them and threw it at me, and stamped at me. I had done nothing to give her any offence. At another time, I took out the saucepan with some potatoes, as she would not have them on the table, and when in the act of giving her the potatoes she took up a poker, when being frightened I ran to the door. She held the poker to me, and stared at me. She said she did not mean to strike me. I then said, why did you take the poker? She replied, "To give it to you, madam, to poke the fire." — Have you heard her say anything about Mary Rainey? Yes, when Mary came up with a turkey, she said when Mary was gone, she only came sneaking for wine. On one occasion, when she was very bad, we would keep the knives from her, but on one occasion she took the knives under her arm, and said, so help her God, she would not part with them. After being with her three weeks, I left, feeling very frightened, and not thinking it safe to be with her.

Cross-examined. I had never been in service before. My little sister had been in the place about a fortnight when my mother took me there. I saw my mother, perhaps, more than once a week when I was there. My mother was an intimate friend of Mary Rainey's. — When was it you told your mother she stamped at you, and frightened you with the poker, and gnashed her teeth. The first time after she did it. — I believe you stopped there till you were discharged? Yes. Mrs. Cumming discharged me. I refused to go at first until my wages were paid. My wages were 8*l.* a-year. Mrs. Cumming was going to pay me 1*l.* for the month, instead of my stopping a month, but took it back again, and said she would pay my mother, and when my mother came up, she paid my mother 15*s.* Mary Rainey and Mrs. Cumming quarrelled about everything. — Was Mary Rainey a very mild person? No, not very violent, she is rather passionate. — You say she took the poker and held it up; where was she, standing in the room? Sitting by the fire. — Did she not say she took that up to stir the fire? Yes. — And did you not take it from her? Yes. — Did you not stir the fire? Yes. — And did you not put it down again? Yes. — Is that all that happened? Yes. She conversed with me mildly and quietly about religion. — She never struck you? No. — You told all this to your mother about the stamping and staring? Yes. — And she left you there till you were discharged? Yes.

By the Commissioner.— Mrs. Cumming would give Mary Rainey money to buy things. Sometimes she would give her a note to change. She had brandy and water two or three times a day.

*Simeon Thorne*, examined.—I am an attorney, in Berners-street. On the 15th of November, 1850, a person came to me from Mrs. Cumming, desiring me to call upon her in Queen's-road. I had an interview with her. She appeared exceedingly infirm. The room was very offensive, it was quite devoid of all atmospheric air. I observed a vast number of cats in the room, probably there were five or six. She wished to consult me on her affairs. She told me she had employed Mr. Robert Haynes, and she stated to me the manner in which she had become acquainted with him. She related that she had had a commission of lunacy against her, and that she had been examined before the Commissioner, and that on that occasion Mr. Haynes was present casually, and undertook her case for her with her sanction. She then complained that she could not get any money from him, that he had had many thousand pounds of her money. I asked her if she had ever had any account. She wished me to remove the papers out of his hands. She said that Mr. Robert Haynes had all the deeds and papers, and that she could give me no information of what her property consisted. On the 21st of November, I saw Mr. Haynes. I applied for his bill of costs. He objected to furnish any account, as it had been furnished over and over again. He said she owed him 500*l.* or 600*l.* for costs; and if I wished to remove the papers from his hands, it must be upon the usual understanding to pay what should be found due to him. He said it would take some time to make out his bill of costs. (Some correspondence between Mr. Thorne, Mr. Haynes, and Mrs. Cumming, relating to a meeting and the accounts, was then read.) Mrs. Cumming's letter, in her own handwriting, was as follows—"Mrs. Cumming presents her compliments to Mr. Thorne, and will thank him to call at 59, Queen's-road, Regent's-park, between the hours of five or six o'clock to-morrow evening." On the 23rd of November, 1850, there is an entry in my attendance-book; "Mrs. Cumming—Attended you by appointment at your house, when you related the circumstances of your case, and the state of your affairs, and particularly as regards Messrs. Robinson and Haynes'

management of same; and it was determined I should apply again for their account and bill of costs, and afterwards see you thereon." I alluded to the account which had been sent. She denied having received a sum of 79*l.* stated in that account. (The witness then went into a long detail of conversations with Mrs. Cumming relating to her property. The points referred to all form the subject of inquiry in Mrs. Cumming's personal examination by the Commissioner.)

(During Mr. Thorne's examination Mrs. Cumming came into the Court.)

The COMMISSIONER, addressing Mrs. Cumming.—Do you know this gentleman (pointing to Mr. Thorne). I saw him once, sir; he intruded himself upon me before I came down stairs. — Are we speaking of the same person? I mentioned his name. (At the request of the Commissioner, the witness held up his hand.)

A *Juryman.*—What is his name, Mrs. Cumming? Thorne, sir. He has been at my house at various times, and I might not have seen him.

The COMMISSIONER.—You are infirm, and obliged to see people in your bed-room?

*Mrs. Cumming.*—Yes.

*Witness.*—Sometimes she was quite unable to give me any account whatever; on these occasions I have noticed she was too ill, merely expressing that she wished to see Mr. Haynes.

*Mrs. Cumming.*—Yes, I was too ill, and no gentleman would have obtruded himself upon me in that state, and I do not go behind his back to say so.

*Witness* continued.—I had a long conversation with her on the 11th of December. She said she had found a will. She gave it to me at the time. There are some red ink alterations. I believe in Mr. Robert Haynes' handwriting.

*A Juror.*—It is not signed.

Sir F. THESIGER.—No; it is the mere draft of a will.

You were going on to state, that you called her attention to the fact of there being nothing in her will in favour of her family? I asked her if it was her wish to leave those legacies in that way. "Certainly not," she said; she had revoked the whole of the legacies to Mr. Robert Haynes.

*Mrs. Cumming.*—That is a falsity.

The *Witness.*—I am very clear upon the subject. I wished to take instructions, as it was her wish I should make her will. *I wished to take her instructions for the purpose of making that will.* — With reference to her family, what did she say? I was then proceeding to say, that I asked her how she wished to dispose of her property. *I was now taking her instructions for her will.* I said you can easily revoke that will. We can easily have another will executed the moment it is your desire. *You are in a fit state of health.* She said she would revoke her legacies to all the Haynes'. When I came to Miss Hunt, I asked who Miss Hunt was. She said Miss Hunt was an old acquaintance, she would wish to leave her a legacy. Would she leave anything to her relations? She immediately became exceedingly excited, and said; "Never mention their names to me, never mention them again, sir," How would you give your property? "To any one who is kind to me," she said. However, I could take no instructions for a will. — She was not in a state at that time to give you any instructions for a will? Certainly not. On the 20th of January, 1851, I saw her about the house, Herbert Villa, which she had taken. She sent to me. It might have been the coachman, or Mary Rainey, who came for me. She had entered into all the matters without consulting me. The agreement was signed by Sir Matthew Wyatt, and *witnessed by me.* On the 28th of January, I stated to her, among other things, that Mr. Haynes had said, that instead of anything being due from him, there was a balance of 500*l.* or 600*l.* due to him. *She rose up in her seat* in a state of excitement, and said; "It is a vile conspiracy." Mrs. Hutchinson was there, and she was evidently under her influence, and she sat down and was quiet.

*Mrs. Cumming.*—That cannot be true, I could not have got up from my seat, because I was in bed.

*Witness.*—I did say there was one exception, when I did not see her in her bed-room, it was in Howley-place. She sat down, and it struck me she was still under the influence of Mrs. Hutchinson, or of something she had been partaking of, and that it would be perfectly useless entering into business on that occasion.

*Mrs. Cumming.*—It is very unmanly in a man saying that, when he is, perhaps, in the habit of doing it himself.

*Witness.*—I remember Mrs. Rainey coming to my offices, and giving me information of what had occurred on the 3d of February at Herbert Villa. I went up to the house.

I could not gain admission. The furniture was being removed. A servant called upon me, and I desired her to make inquiries where Mrs. Cumming had removed to, and through her I learned that she had been taken to 104, Stamford-street. I went there and could not get admission. I afterwards got a note signed by Mrs. Cumming, desiring me not to call again or trouble myself about her affairs. In the month of November last, I heard she was in Effra Hall Asylum. I called on her there. I was permitted to see her in the presence of Mr. Elliott, the proprietor. She said, "I do not want to see you; leave my room." She said I had deceived her, and wronged her. "I do not want to hear you, I won't hear a word." She refused to have any communication with me. The attendant said, "Do not excite yourself." She then said, "If you can get me some money that is a different thing, that is the point." I said, "If I was allowed time, that I dare say I could get her some money for her property." Then she said again, "Then I will talk to you."

Cross-examined.—I was introduced to Mrs. Cumming by Mr. Fase, my brother-in-law. He is a silversmith. She had applied to Mr. Fase to recommend her a solicitor. When I went, it was Todd who let me in. Mrs. Cumming appeared to be in an extremely debilitated state. There was a total loss of mental capacity. — Show me the entries (in witness's book) where you begin your transactions with Mrs. Cumming. Friday, 15th of November, 1850. This is the first entry; "Attending you, and conference on your affairs, and I was to apply to Robert Haynes, solicitor, for the account of the rents, &c. received by him." Yes. — That was the entry you made in reference to a person who appeared to have no mental capacity? — Do you recollect producing to Mr. Haynes Mrs. Cumming's written authority? Yes, afterwards, and I will tell you why I did. The reason was for my ulterior proceedings, and having some doubts about Mrs. Cumming's state of mind, I thought it right to have something under her hand. — Now will you explain what the production of Mrs. Cumming's written authority to Mr. Haynes could possibly have to do with your doubts as to her state of mind; because, if you thought her of unsound mind, what was the use of her written authority? In point of law it would be none. — What reason was there why you should show that to Mr. Haynes? I can see no other reason, than that I required in a proper manner all deeds and papers I had previously written to him for. — The reason you urged just now was, that you had some doubts of Mrs. Cumming's soundness of mind. "The bull put his head out of the window, and so she died, and he married the gardener." — What possible connexion can there be between your showing that to Mr. Haynes and your doubts of the lady's soundness of mind? I conceived the question as such, and one that would be better answered by the jury by their verdict in this case. Whether right or wrong I chose to get it, and I did that.—When was it that the circumstance took place with regard to taking the house? The 21st of January, 1851. — Did you attest that agreement? Yes. — Mrs. Cumming being a party executing it? Certainly.

(Serjeant Wilkins then requested the witness to read the subsequent entries of his attendances and conferences with Mrs. Cumming, some of which will be found in his examination in chief.)

It was on the 21st of October, 1851, that I went to Effra Hall.—Did any one go with you? "Having ascertained that Mr. Turner, solicitor, was acquainted with your present place of residence; writing to him requesting to be informed thereof in order to obtain an interview." Upon which I and Mr. Turner went together. — When did you first correspond with Mr. Turner? On July 1st, 1851. — When did you first see Mr. Ince, or Mr. Hooper? I never saw Mr. Hooper at all, to my knowledge, until I saw him here. Mr. Ince called about the time, after the removal of the goods at Paddington (Herbert Villa) in February. — Will you look and see whether there is any entry of your interview with Mr. Ince? On the 15th of February Mr. Ince called on me, and I charge it to Mrs. Cumming. On the 6th of March Mr. Ince came to me again, "Conferring respecting Mrs. Cumming, and your determination to proceed with the Commission of Lunacy." — In your affidavit you say, "that on the following day I went to Herbert Villa aforesaid." You told us yesterday you were refused admittance. Were you not told, at the same time, that Mrs. Cumming was very ill in bed, and could not be seen? Yes, the person said she was too ill to be seen. You say in your affidavit, that you frequently, since the 12th of February, had seen the late servants of Mrs. Cumming, and that you had endeavoured through them to discover where she resided. By the late servant do you mean Mrs. Rainey? Yes, and Mrs. Hickey.

By the COMMISSIONER.—Would you as a professional man have taken instructions from her to make a will? I think not. — If she had given you instructions? If she had, *I should have made a will merely revoking what she had done.* — If she had given you instructions would you have felt justified in carrying them out? I certainly should have had medical advice; and I mentioned it to Dr. Caldwell that it would be necessary probably for her to make a will, and that I should like some other gentleman to be present.

*Nathaniel Webb,* examined.—A surveyor from Newport. Knows the property of Mrs. Cumming in Monmouthshire. This witness described one property in the parish of Bettws as having been going to decay for twenty years; he considered it would cost 300*l.* to put it in repair. If in proper repair it would be worth 45*l.* a-year. In the present state of repair of the buildings it is not worth much more than 32*l.* a-year, which it now produces. Another farm, in Cwn Bassaleg, would require 460*l.* to put it in repair; the house is a complete ruin. The rent is 60*l.* a-year. If 460*l.* were spent upon it, it would produce 95*l.* a-year. Another farm, called David the Clerk's Farm, of 33 acres, if sold for building at twenty years purchase would exceed 12,000*l.* It has been sold to Sir Charles Morgan and Mr. Bailey for 2140*l.*, being the average of 65*l.* an acre. The witness described other farms as being also in bad cultivation. There is a farm called the Blackbird's Nest, I cannot give you any account of that, I merely walked by the house; that land looked in good condition; I did not trouble much about it. The witness mentioned also that a house had been sold for the station of the South Wales Railway; that another, at Stow Hill, had been sold to the Water Works Company.

Cross-examined.—The land sold to Messrs. Bailey and Morgan had been first offered by auction in six lots, and advertised for building purposes, and bought in; it was then sold by private contract. At first said the average rent of pasture land in the neighbourhood was 50*s.* an acre, afterwards that he could not tell. Some of the buildings are reputed to be 150 years old. Most have been in the same condition for the last twenty years.

*Mrs. Ince,* examined.—The youngest daughter of Mrs. Cumming; married Mr. Ince in 1833, with the approbation of both parents; at that time lived on the best terms with my mother. I remember my sister's marriage with Mr. Hooper; it was not with the approbation of my mother, nor of any, in fact. I took every means to prevent it. Three years after the marriage, my sister was reconciled to her mother when the second child was born. From that time my mother was affectionate towards Mrs. Hooper. At different times after that period there was a strangeness about her: little things she would make a great deal of. About 1838 or 1839 she was very much changed, very much altered in her manner and in her conversation. Upon one occasion she asked me to dine, with an infant I then had. I was about ten minutes past the time; she asked me what brought me there, I said she had invited me to dinner; she said, "Yes, I invited you at five, and it is now ten minutes past." I pleaded my infant as an excuse. She said, "If people do not come to my hours, they do not dine with me." She allowed me to go. I thought that strange, and contrary to her usual manner. From that time to 1845 or 1846 she exhibited at times a strangeness in her manner towards myself and my sister, and other persons. It seemed a pleasure to her when with one daughter to say something against the other. She would stay away from me for a year. When my sister was friends with her she was not friends with me. My sister thought it was as well, as mamma was so odd, that I should not see her. I heard of her from my sister. Both myself and my husband treated her with the greatest kindness. I remember my father and mother living in Belgrave-place, in 1846. My father was very old; my husband used to send him brandy in physic bottles. Once my father came to our house for protection; he said that my mother's conduct was such that he could not remain. I remember my mother being removed to York House, in 1846. My sister and myself were not the instituters of that proceeding. My father thought it to be the best step to be taken, and my sister and myself signed the paper to satisfy his mind that the act was right. — Do you entertain any doubt it was right? I have not the slightest reason to regret it, only to regret that the arrangement was not carried out at the time. — After your father died, you and your husband and Mr. and Mrs. Hooper continued the Commission of Lunacy against her? Yes, we had the Commission held to give her the chance of proving whether our judgment was right or wrong.

(*Vide* Mr. Dangerfield's evidence, who says the Commission was not taken out until the Commissioners in Lunacy had been applied to to appoint a receiver of the estates, and it had been discovered that they had no such power.) After the Commission, my mother was taken to Mrs. Hutchinson's. I called, with my sister, at Mr. Hutchinson's; he refused to give me any information about my mother. After some time, we found she was at St. John's-wood, and then I went to see her. We traced her to Camberwell. It must have been three years that I lost sight of my mother. I learned from Mrs. Hickey that my mother was about being removed from Herbert Villa. I did not go there; my husband went and saw Sir Matthew Wyatt. Afterwards I learnt, I think from Mrs. Hickey, that Mrs. Cumming was in Stamford street, in the house of Mr. Hutchinson. I went there with a gentleman; we were refused admission. Afterwards I was told she was in the Edgware-road; that was told me by Mary Rainey, I think. It was the house of Mr. Oldfield. I went there. It was on the 26th of May last. The servant said, "She does not live here." I said, "Excuse me, I am certain she does," and I passed up stairs. I saw my mother sitting at the window, seeming to be at lunch. Mrs. Oldfield followed, and asked me, "What business I had there?" My mother was very feeble. I asked her if I should cut the meat for her, for which she thanked me, and I did so. Mrs. Oldfield sent for Mr. Haynes; when Mr. Haynes came, he said, "What business have you here?" I said, affection brought me, and duty to my mother. I appealed to my mother whether I should leave, she said, "No, my child, I will never do that." I spoke to her about her will; I said that I was quite aware that her property was given by her will to Mr. Robert Haynes, and that I had no wish but to see her comfort studied while she remained in this world. She said, "Not my will, and that I have told Mr. Robert Haynes." I was there three hours. Mr. Haynes did not leave, and I left. I went again the next day; Mrs. Oldfield opened the door, I followed her up stairs. My mother was partly dressed; she asked me to assist her; then Mrs. Oldfield screamed and stamped, and clapped her hands, and frightened my mother. It brought the servant up. On the following day, I called with my sister at the same place; a note, signed by my mother, was put out at the door. (It signified Mrs. Cumming's request that Mrs. Ince and Mrs. Hooper should not be admitted.) I afterwards learned that my mother had gone to Worthing, and then to Brighton. This Commission is presented by myself and my husband and Mr. and Mrs. Hooper, for the purpose of protecting my mother's person and property. My husband is too ill to attend here; he has not left his bed for four days.

Mr. Serjeant Wilkins.—For reasons which will be obvious to every one, I should wish to postpone the cross-examination of this lady until it be definitely ascertained whether Mr. Ince will be here, because there are many questions that I should wish to avoid putting to her which I should put to Mr. Ince.

Sir F. Thesiger.—From what I hear, he is so unwell I do not think he will be able to attend.

(A discussion then took place: Mr. Serjeant Wilkins contended that the Commission ought to be adjourned until Mr. Ince, who had made important affidavits in support of the Commission, could be produced. Sir F. Thesiger replied, that he was only bound to call what witnesses he thought fit. The Commissioner decided, that the learned counsel must take such course as to the cross-examination as he thought fit; what might take place afterwards was another question. A Juror observed, that Sir F. Thesiger in opening the case pledged himself to produce Mr. Ince. Mr. Serjeant Wilkins then applied to have the cross-examination of Mrs. Ince postponed, to see whether Mr. Ince would come or not, as in the case of his coming he should not ask Mrs. Ince half-a-dozen questions. Sir F. Thesiger refused to assent to the postponement. Mr. Serjeant Wilkins ultimately proceeded to cross-examine.)

*Mrs. Ince*, cross-examined.—At the time of my marriage, my mother was very social. She frequently attended and gave dinner and evening parties; they gradually ceased after I married. She kept up an acquaintance with Mr. and Mrs. Hutchinson. Miss Hunt was her dress-maker. I do not know that she made a sort of confidante of Miss Hunt. Mr. Driver, who was our assistant, used to visit her. When I was living at home, my mother was not in the habit of drinking ardent spirits. I remember an execution being put into my mother's house; I heard for my father's debt—a gambling debt, or a debt of honour. I heard that he afterwards took the benefit of the Insolvent Debtors' Act. I stayed with my mother a month at Greenwich, in 1837. I think in 1840 or 1841 was my next visit to her; it was at Maida Vale. I might be there two or three weeks. I could not remain, her conduct was so strange; in fact, I was so ill I was compelled to go home. I did not see my mother again until she came into our neighbourhood, about 1846. In the interval she came to the funeral of the little boy that died. She saw the child after it was dead, and she made remarks about its being glazed. I was at her house after that; we were invited with Mr. Driver; there was a supper, and then she was strange and excited, and, in fact, it broke up the party. I cannot name any other occasion from 1841 to 1846 of my seeing her. — Will you allow me to ask how many attorneys you have had in reference to the transactions with your mother? — The witness enumerated Mr. Dangerfield, Mr. Turnley, Mr. Jones, and Mr. Turner. I heard from Mr. William Jones, as well as Mr. Ebenezer Jones, that a charge of perjury was preferred against my mother. I heard of it as being contemplated; I refused my sanction to it. I know that Mr. Ebenezer Jones on the former occasion was produced to prove my mother was of sound mind. — Have you seen him here? I saw him at the door. — Do you not know he is subpœnaed as a witness on your side? No, I am not aware of it. He was several times at our house on this subject. It may be a week ago he was there. Whenever he has come I have never refused him admission; I always asked him to take refreshment. He may have dined there two or three days in the week. Mr. Jones may have been at our house the day before my mother was apprehended on the charge of perjury. I went to Mrs. Hutchinson, in Stamford-street, to let her know I had nothing to do with this matter. I was refused admission. I will not state positively that Ebenezer Jones was not at our house on the very day my mother was apprehended. I am satisfied not having seen Mr. Jones from our disagreement till after all proceedings had gone by. I think it was Mary Rainey who told me my mother had been arrested. Mrs. Hooper was married from our house. I went to the church with my sister. My mother refused my sister admission into the house. Mr. Ince took the licence. She came back to me and claimed protection. I said I could not keep her there; it was contrary to my mother's wishes, as well as my own.

Re-examined.—It was a very melancholy marriage altogether. We did our best to prevent it. The witness then detailed the different residences of her mother down to 1846. There were some glass salt cellars that a servant had robbed them of and pledged. She requested Mr. Ince to redeem these articles. I offered her to take them in the carriage, and she refused. A policeman came one morning and demanded the property of Mr. Cumming. This was while she was at Greenwich, in 1837. The men took them away. After the execution, the things were put up for sale. Amongst the articles of plate and jewellery, was a silver basket. Mr. Ince wrote to my mother, offering to buy it in. We attended the sale, not receiving any reply to our letter. We took on ourselves to purchase the basket. We wrote to her to tell her the basket was at her service. She made no reply. Then, among other things, a time after, she asserted that we had stolen this silver basket; eventually she had the basket. I was never at Herbert Villa at all.

Cross-examined.—When your mother saw the child in the coffin, did she not say it looked like a little wax doll? No: a remark was made that it was glazed over, and that Mr. Ince had done so; but she said it looked like a doll in a tailor's shop; that was the remark she made. I certainly must admit that I was reluctant to part with the silver basket, because I understood there was such a bad set of people about her. It might be a year or two before it was sent to her.

*Mr. Wilmot* was here called, to speak to the illness of Mr. Ince. He is in a very precarious state of health; he is suffering from nervous depression, and is threatened with paralysis; he has partially lost the sight of one eye; I am afraid he will not be able to attend for some time. — He is threatened with paralysis? Yes; he has partial paralysis in one eye.

Cross-examined.—When was it you were first called in to see him? I am speaking from memory, but I should say, six or seven days ago. — When was it his illness began? I think it was about six days ago. — When did you communicate this to Mr. Turner? Did you not hear my friend open the case, and say he would call Mr. Ince? No, I did not. — Mr. Turner has been in communication with and seen Mr. Ince. I probably mentioned it to Mr. Turner on the first day of the inquiry. I am not sure of it; it may have been on the second day. (The witness was speaking on the *fourth* day.)

Sir F. Thesiger: I am bound to say, I certainly did pledge myself to produce Mr. Ince, and I intended to do so.

*Daniel Pilditch*, examined.—This witness was a builder, called to prove the value of the two houses in the Queen's-road, purchased by Mrs. Cumming of Mr. Haynes. He valued them at about 1100*l*. On cross-examination, he admitted he had never built or valued any houses in the neighbourhood of Regent's Park. He calculated that house property ought to pay 10*l*. per cent. His evidence has no bearing upon the sanity of Mrs. Cumming.

*Mr. Benjamin Bailey Hooper*, examined.—I am one of the sons-in-law of Mrs. Cumming. I was married to the eldest daughter in 1836. At the time of the marriage I was a commissioned officer in her Majesty's Excise. Before I was in the Customs I was educated for the profession of music. I belonged to one of the Guards' bands. I had not visited at Mrs. Cumming's house before my marriage. I had known her by passing and re-passing in the street. My marriage was without the consent of Mrs. Cumming. I was married from Mr. Ince's house. Mr. Ince was present at the marriage. I informed Mrs. Cumming by letter of the marriage. We were not reconciled for some time; I think it was in 1839, on the birth of the second child. I wrote to Mrs. Cumming, to inform her of that event, and my wife received a reply from her. I think, to the best of my recollection, couched in something of this sort—that she was very happy to hear—the usual thing—and that the child thus born should be a peace-maker. In November, in that year, I was invited to my father and mother-in-law's, but previous to that, my wife had been to her mother, immediately after her confinement, and was received very affectionately; I was not present; on the occasion when I was invited to dine, I was received with great kindness. Early in 1840, a little disagreement arose. My wife said to her mother, at her mother's house, that she missed a pocket-handkerchief. Mrs. Cumming flew into a violent passion with my wife; she said something about believing her servants in preference to her daughter. We left the house immediately. This produced an estrangement for some time. In the summer of 1841, Mrs. Cumming came to our house again; her former feelings seemed to have returned towards her daughter. She invited her daughter and myself to stay with her several times. I did not exactly stay myself, but my wife did. I went as my business would allow me to go. In September, 1842, I remember Mrs. Cumming sending a little girl to my house, requesting me to go in search of Captain Cumming; that he had left his home, and she did not know what had become of him. I went to her; she was then living at Maida Vale. I arrived in time to see her alight from a cab in company with Captain Cumming. She passed me without any recognition—did not seem to know me at all. I followed her, and when she got to the door, she turned round and stared at me in a very particular sort of way. "What do you want? the sooner you take yourself off the better." In 1843, I wrote to her, and went down to her house with my wife. She knew me, and received me kindly. I then saw her from time to time until her removal to Belgrave-place. I was consulted by her, about the end of 1845, about the management of her property. At her request I agreed to be her agent. Receipts were prepared for me to go into Monmouthshire to receive her rents. I had that authority signed by her at that time. It is in my hand-writing. I called upon her to receive her final instructions; she refused to see me. This was in July, 1845; no reason was assigned for her refusing to see me. In 1846, I remember Captain Cumming coming to me to take shelter from the alleged violence of his wife. He remained five or six hours; I went back with him to the door; he afterwards came again and took refuge: he remained two days and two nights. I went back with him on that occasion; I saw Mrs. Cumming; she met us at the door. She exclaimed (alluding to the captain) "I would rather see him brought back dead than brought back by such a scoundrel as you." There had not been the slightest quarrel between us before that. I had not seen her since the time when she gave me authority to collect her rents. The commission was issued in 1846, with my sanction, my wife's, and Mr. and Mrs. Ince's. When that inquiry took place my wife was in a bad state of health and unable to attend. She was not at the Horns' tavern at all. From 1846 down to 1851 I did not know where Mrs. Cumming was at all. I endeavoured at different times to discover, but always failed.

Cross-examined.—I used to play in the theatres; in the orchestras. I have played at all the theatres, at Astley's. It is correct that I was a bandsman, and played at Astley's. There is no delusion as to that. I was in the band when I first paid my addresses to Miss Cumming. The first time I met her was at the chapel door, on Sunday evening. I used to walk backwards and forwards in regimentals. I met her sometimes on Sundays. It was three years after my marriage before I saw Mrs. Cumming. The family might call me the bandsman. I used to play the French horn.

Before Mrs. Cumming signed the authority for me to collect her rents, I had asked her to lend me money. — Did you ask her to be a party to raising money upon your wife's reversionary interest? Not to the best of my recollection. — Did you apply to Mrs. Cumming to be a party to it? I do not remember it. I might have spoken on the subject to her.

I signed the petition in 1846, that Mrs. Cumming was a lunatic at that time. I am aware of the arrangement that was made. After that arrangement we changed our attorney, and employed Mr. Turnley. In 1848 I gave instructions to Mr. Turnley to file a bill in Chancery against Mrs. Cumming. There was a friendly decree by which the suit was arranged, and I received a considerable sum of money. A sum of 1000*l*. was raised to pay Mr. Turnley's bill. I should have 60*l*. a year under the decree. Those are the terms of the compromise of the suit in 1848.

You have sworn to Sir Frederick Thesiger that, from 1846 to 1851, you did not know Mrs. Cumming's address. I ask you, Mr. Hooper? I think I was mistaken there. I did hear she had been living in this neighbourhood, I think. — Will you swear you did not know it from 1846 down to the end of 1849? I will not be sure about it. — Did you know from Mr. Turnley that she made affidavits, and that every affidavit set out her address from 1847 to 1849? I did not. — Did you ever ask him for her address? I do not recollect that I did. — Did you know her address in 1850? I think I did. My wife called on her in 1850.—Where was it? It was up here. — Now, I ask you, when you got the money in 1848, under the compromise of the suit in Equity, you did not apply to Mr. Turnley again to institute a Commission in Lunacy; and whether Mr. Turnley did not refuse, stating it was arranged that she was to be treated as a sane person, and that he would be no party to it? No. — You did not? No. — You swear that? Some conversation of the kind might have taken place; but Mr. Turnley was never instructed to do anything of that sort.—What was the conversation? A conversation whether there were any grounds for doing it. — I know Ebenezer Jones; he was examined at the former commission, upon the part of Mrs. Cumming, to prove her sane. I have seen him here to-day. Very busy with your witnesses, I believe?

Sir F. Thesiger.—If he is, we are not answerable for that. When he comes, if he does come, you may ask him that question.

Mr. James.—You say, "If he does come."

Sir F. Thesiger.—I do say *if*, because I do not mean to call him.

Mr. James.—He has made an affidavit, upon which the commission is founded.

The Commissioner.—He is here, I understand.

I knew of my mother-in-law being arrested on a charge of perjury after it was done. It may have been in 1849 that the money was raised under the decree of 1848, and that I got the 300*l*. I do not recollect. I had seen Jones before Mrs. Cumming was arrested by him. He was at my house once. It may have been a week, it may have been a fortnight, before the arrest that I met him. I will not swear I had not seen him the day before. I saw him at Mr. Turnley's office on the very day. I met him there by accident. The arrest was spoken of. I did hear Jones say something about going to arrest the old lady upon a charge of perjury. I think I was at home when she was arrested. I might have seen Jones the next day. I do not know where. I first heard of the arrest in Mr. Turnley's office. I have played a game of whist with Jones, and shall do again when I want to make up a party of four. I knew Captain Cumming when he took the benefit of the Insolvent Act. I am aware of the execution. He was not a violent person. I never heard him swear.

Re-examined.—I had nothing to do with the arrest of Mrs. Cumming. I endeavoured to induce Mr. Jones not to do it. I considered she was not in that state of mind that she was accountable for her actions.

Further cross-examined.—You just now stated, that in your opinion she was in that state of mind at the time of the arrest that she was not answerable for her acts. Had you seen her since 1848, when the arrangement was made under which you got the money? No. — Had you seen her since 1846, when an arrangement was made to quash the Commission? I saw her once in the park. I did not speak to her, — And yet you state to the jury she was not in a state to be responsible for her acts? Yes.

Re-examined.—Almost from the first interview I had with Mrs. Cumming I thought she was of feeble mind. The impression left on my mind in 1846 remained.

*John Turner* examined.—The solicitor for the prosecutors of this proceeding. I first became concerned for Mr. Ince in the beginning of January, 1851. From facts which had been communicated to me by Mr. Ince and Mr. Hooper, I called upon Mr.

Thorne in June, 1851. I asked him for information as to where Mrs. Cumming was to be found. I went direct from him to Mr. Robert Haynes. He refused to give me Mrs. Cumming's address. In consequence of this refusal, sometime subsequently I applied to the Lunacy Commissioners to see if they could give any information. In the interval I saw the late servants, and requested them to make inquiries. About October last, I obtained information from the Commissioners that she was at Worthing under the name of Cleveland. I sent a detective officer to Worthing, and I learnt that she had gone to Brighton. At this time, it was finally determined that we would get a commission, but that Mr. and Mrs. Ince should present a petition to the Lord Chancellor for a medical examination. A petition was presented, and an order obtained, having Dr. Monro and Sir A. Morison to make that examination.

(A discussion hereupon ensued between counsel as to whether the order of the Lord Chancellor should be read. Mrs. Cumming's counsel contended that it was not evidence, being obtained upon *ex-parte* statements, and might prejudice the case: also, that it was obtained chiefly on the affidavit of Ebenezer Jones, whom Sir F. Thesiger stated he would not call. The Commissioner said that the contents of the order were no evidence; the order itself might be a different affair. Ultimately the order only was read, and not the petition which was recited in the order.)

The following is the order as read:—"I do hereby order that Dr. E. Thomas Monro and Sir Alexander Morison be at liberty either alone or jointly, with any person or persons they may think fit, and in such respects and under such regulations, and at such times as they or either of them may deem necessary, to visit and examine the said Catherine Cumming for the purpose of ascertaining the state of her mind, and her competency to manage herself and her affairs. And the said Doctors Monro and Sir Alexander Morison, are to certify to me in writing the result of their examination and opinion as to the state of mind of the said Catherine Cumming, and the grounds upon which they form such opinion, after which such order shall be made as shall be just. And I do hereby order, that all persons be and they are hereby restrained from interfering with or interrupting, or causing to be interfered with or interrupted, the said Doctors Monro and Sir A. Morison in such visits and examinations aforesaid. And I do hereby further order, that all persons be and they are hereby restrained from removing the said Catherine Cumming out of the jurisdiction of that part of Great Britain called England till my further order.

(Signed) "TRURO."

(We quote the order in full, as under the assumed authority conferred by it, certain proceedings were adopted, the propriety of which was questioned. These will appear in the subsequent evidence.)

Having obtained this order, I first went down to Brighton. I went to the chief officer of police and told him of the order I had obtained, and of the abduction, for so I termed it. I made application to him to prevent any breach of the peace if any such should be attempted. On Monday, the chief officer of police went first and gained admission to the house. I then went and found the officer had got admission. I saw no one. The officer let me in. I then went to look for Sir Alexander Morison and Dr. King, and when I returned, I found Mr. Robert Haynes, and a Mr. James. I served Mr. Haynes with the order. We requested that we might be allowed to see Mrs. Cumming. He seemed quite unwilling to give us any assistance. Mrs. Ince was with us. There was a long time elapsed in conversations and persuasions by the doctors, and eventually, after both Mr. James and Mr. Haynes had not replied, I said either this must go for nothing, and the Chancellor's order must be treated as waste paper, or the door must be opened. The chief officer asked me when I conceived a reasonable time had elapsed; we thought two hours was a reasonable time. He then forced the door. Mr. Haynes came forward and threatened every one, and said he would take proceedings. We ultimately gained access to Mrs. Cumming. Upon the door being forced open, Mr. Jones stood before it and tried to prevent the physicians going in. The officer was in front. Sir A. Morison said he did not wish anybody to be present with him save myself. Mr. Jones endeavoured to force his way in. The officer stopped him. Sir A. Morison, myself, and the servant, Mrs. Watson, were present with Mrs. Cumming. I made a memorandum of what passed in Mrs. Cumming's presence. Sir Alexander Morison stated to Mrs. Cumming, that the Lord Chancellor having heard that she was very unhappy, had requested him to seek her, and he had to question her by his lordship's desire. She raved at first, that is, she was vehement in her manner, but after a time became more quiet. She was asked if she would not see her daughter, and shouted, "No, never." She was asked why, and said she had tried to murder her. She was asked when, and she said at Howley Villa; Catherine Ince tried to strangle her, and she screamed murder. That her children robbed her, and she could prove it. That Mrs. Hickey wanted to take her to a madhouse, and her daughters wished to poison her. The servants had tried to poison her. She knew poison was put in her tea-cup, and she could prove it by medical men. She left her house in the Queen's-road because the servants tried to poison her; and she was continually moving about for fear of her daughters, who wished to kill her. She was asked if she had made a will, and said, "Yes," but she did not know when. She was asked who she had left her property to, and said, to her benefactors, who had protected her from her daughters. She refused to name them. She was asked if she knew Mr. Ebenezer Jones, and replied, "Do I know the Diable," that fellow has robbed me. She was asked of what, but could not tell. Had he been her agent? She replied, "Yes," she had employed him through Mr. Haynes, but she had never made any affidavit about him. She had not sold any of her property, but was living upon it. She could not tell the value, or of what it consisted. She was asked whether her son, Mr. Ince, had not attended Mr. Cumming, and shouted out, "Do not call him my son, he is the greatest villain that ever lived." She disapproved of her daughter's marriage, and, so help her God, would never see her again. She was asked if she knew Mr. Thorne, and answered, with vehemence, "Yes, that villain robbed me." I interposed at this time, and assured her that he was a gentleman holding an official situation; she said, he may be *officially* honest, but he had robbed her. She was questioned as to her grandchildren, and did not know how many she had; she hated her children, and felt no interest in her grandchildren. Mrs. Ince was a very bad character; she had seen her surrounded by six policemen, in a dreadful state, and beastly drunk. That was Sir Alexander Morison's examination.

Then Dr. King asked her if she was married, and she indignantly answered, she considered the question impertinent. She again refused to see Mrs. Ince. She was asked why she employed Mr. Thorne, and said, to do the same things as Mr. Haynes. She was questioned as to her property, but could give no account of it. She was asked if she had houses in the Queen's-road, and answered, Yes; but she could not tell what they cost, and she bought them of Mr. Haynes; she knew they were mortgaged. She did not know the rental of her property. She said she had sold the Red-house property, but did not receive the money. She did not know what property had been sold, nor could she state for what; she did not know why she had sold her property. Every possible assurance was given her of her daughters' affection for her. It was tried by both doctors to induce her to see her daughters, but she was deaf to all persuasion. She forgave them, but she could never forget. She felt great pain in body; she had fits. After her daughter had endeavoured to strangle her, a man and woman were in the house, and she was told if she was not quiet, they would put her in a strait-waistcoat, which they had ready; the man's name was George Clark. Lots of policemen and soldiers were brought into the house. She went to Mrs. Hutchinson's and got out of their hands. She was very fond of cats, and took them in her carriage; and one was a postilion, another a coachman. She was indignant with Dr. King when he thought them a nuisance, and asked, if he doubted her? he perhaps was not fond of cats.

That is the whole of the memorandum; that contains the substance of the examination of Sir A. Morrison and Dr. King. After they had done examining her, I asked if I might put a few questions to her; she gave me, in a very gracious manner, permission to do so. My object was to induce her to see her daughter, and therefore I sifted the answers she had previously given. I have not a memorandum of my questions. I asked her how long it had been since her daughters became so unnatural towards her, and she could not tell me. I took her from her infancy; I said, you have nursed them, you have suckled them, you have seen them as children, when was it that they became these degraded beings that you describe them? She could not tell. Was it before their marriage or afterwards? She could not give any time; she thought it was after their marriage. I questioned also as to where she had seen Mrs. Ince in the state she described; also about Mr. Clark; nothing material was extracted. After this interview, I requested Sir A. Morison and Dr. King, in case Mr. Haynes would not undertake not to remove her, *that they would give certificates so that I might take charge of her*. Having got these certificates in my pocket I went back to her house. I applied to Mr. Haynes to give an undertaking not to remove Mrs. Cumming for a week, and to permit her to be seen in the meantime, and that he refused to do. — Upon that refusal you got the certificates? I had the certificates before. I then saw Mrs. Ince. Mrs. Cumming was left with Mr. Haynes and her own servant; I had an officer there. I telegraphed to town that some one should come down to take charge of her, or otherwise she would be removed. The next morning the nurse came down. I went with her to the house. I told her not to alarm Mrs. Cumming, and, as quietly as she could, remove her to the asylum, at Effra Hall. She was not removed on the Tuesday. Mr. Haynes refused to oppose by force her removal. She was ultimately removed. I was telegraphed of her coming, and I met her on her arrival. We had provided a staff of police in case of any further difficulty in town. I apprehended that Mr. Haynes might attempt to remove her.

Cross examined.—The suggestion for petitioning the Lord Chancellor for a medical examination of Mrs. Cumming originated with me in October. I read the order attentively. — Will you tell me then by what authority you presumed to put any questions to Mrs. Cumming? I had her permission. I went down to Brighton on Saturday, the 24th October. I went to Mr. Chase, the superintendent of police. I think I did accuse Mr. Haynes of abduction. I went to the house on the Monday with the superintendent and Mr. Meates (Mr. Ince's partner). There were two officers in plain clothes, Mrs. Ince, Dr. King, and Sir Alexander Morison—that is eight altogether. When I returned back, the carriage was at the door and Mr. Haynes was in the house; that was about an hour and a quarter after we had first gone; he afterwards told me he had come down to dine with Mrs. Cumming by her invitation for that day. — Did Mr. Haynes say that such an attack on the privacy of Mrs. Cumming was enough to drive even a sane person mad? Certainly not. — Did Mr. Haynes beg that Mrs. Cumming might have sufficient time given her to compose her mind? He asked if I would go away for about four hours and then return. — Did not Mr. Haynes say that if you would give her time to compose herself, that he would give his personal undertaking that she should submit to an examination? I think he said that if we would go away for four hours he would give his undertaking that she should not be removed. — Will you swear that he did not say he would give his undertaking that she would submit to an examination? I cannot swear one way or the other; my impression is, he wanted us to go away for four hours. It was at my request that Mr. Chase burst open the door. When I and Dr. Morison went into the room, Mr. Haynes had asked to be present. Sir A. Morison objected. I myself put several questions to Mrs. Cumming; I cannot say, a great many. I swear that I did not ask her whether she had seen Captain Cumming in the act of copulation with one of his servants; no one in my hearing used any such offensive expressions to her. Dr. King asked her about her husband, if he ever did anything improper; she hesitated, and said he was dead, and she said she did not wish to speak of the dead. He then went further and said, nothing improper ever took place between him and any of the servants; she hesitated some time and said, "Oh, yes, he had had a bastard child." On this occasion, I interposed, "That was only once, was it?" and she said in answer, "Oh, yes, I caught him in the act constantly." I altered some of my memorandums the same evening when I got home. Dr. Morison was quite an hour examining her. When Dr. Morison left, Dr. King came on. She was under his examination an hour and a half. — Did it never occur to you that this lengthened examination was rather too much for a lady in her position? It did not occur to me, for I did not think of it, in truth. She had refreshment brought up while I was there. The room appeared clean. There were no cats in the room. Mrs. Watson went out and in; she was out not more than five or six minutes at a time. Mrs. Ince and Mr. Meates left about an hour before I went. I left a police officer in plain clothes all night. The next morning I went to the house with a male and a female keeper. At 4 o'clock Mr. Elliott came. — Now you had obtained an order from the Chancellor for a medical examination? Yes. — And were you not commanded to return the result of that examination to the court? The medical officers were. — Did you in the meantime get a certificate signed by Drs. Morison and King for the removal of this lady to a lunatic asylum? On the Monday after the examination and the obstruction that had taken place, I asked them to give me certificates, in case I should get no undertaking. — Tell me one act of obstruction that was offered by Mr. Haynes or any person there? Mrs. Watson I should say was one, running up stairs and locking Mrs. Cumming in her bed-room. — Did you hear Mrs. Ince ask for admission into the bed-room? Yes; I did. — Did Mrs. Cumming say, "I have commanded her to lock the door, and I expect that she will obey my command" ? She commanded her not to open the door; subsequently we called on Mrs. Watson to open the door; and she said, "I desire you not to open it." — Now, what other act of obstruction was there ? Mr. Haynes, before the door was forced open, saying "If any one forces open the door, I will take proceedings." I might have seen Dr. Hale. I was told by Mr. Elliott that Dr. Hale had certified that to remove her in her then condition would be dangerous. I was not aware that she was paralysed in part of her body. At the time of her examination, she complained of pain in her back, and in her joints, and she felt pain all over. Mr. Elliott told me he should not act without my authority. I authorized him to remove her if it was right; of course if she was unable, she was not to be removed. I was not present when any one said there was a warrant from the Chancellor. I told them in the house that the disobedience to the Chancellor's order exposed them to be sent to Newgate. I did not instruct counsel to say, in answer to an application from Mr. Haynes to be admitted to Mrs. Cumming, that it was not the wish of Mrs. Cumming to employ Mr. Haynes as her attorney. — Did not Mr. Wilde, in answer to that statement; communicate with the Lords Justices ? I heard Mr. Wilde state it was her wish to employ Mr. Haynes. I first became acquainted with Mr. Ebenezer Jones; I instructed him to bring up Webb. I subpœnaed Mr. Ebenezer Jones; I am liable to his expenses for having brought him up.

Re-examined.—The re-examination merely recapitulates some of the circumstances attending the entry into Mrs. Cumming's house at Brighton.

*James Johnson*, examined.—A policeman. Belgrave-terrace, Pimlico, was in my district in May, 1846. Between ten and twelve o'clock in the day my attention was directed to Mrs. Cumming's house. I saw Mr. Ince first in the street. I saw two females who were come to remove her; they came in a fly. I went up to Mrs. Cumming's door and knocked; the door was opened a little way, and one of the females, endeavouring to get in, pushed her knee and leg between the door and jamb. I took my truncheon to ease her foot and leg; that got admission into the house. It was the street door we forced open. I asked these females what authority they had; they produced the certificate. I saw Mrs. Cumming. She said we had come, or were sent, to murder her; I was no policeman. I stood reasoning with her, to persuade her to come. She refused, and made use of some very violent words. They took a strait-jacket from under their shawls, and threw it over and under her chin, and put her arms in across the front of her body and tied it below.

Cross-examined.—I saw Mrs. Cumming afterwards at the Horns' Tavern. I saw her walking up and down quietly in company with two females.

